# Editorial: Global approaches to molecular diagnostics for pediatric cancer

**DOI:** 10.3389/fonc.2025.1578509

**Published:** 2025-03-06

**Authors:** Jeremy R. Wang, Thomas B. Alexander

**Affiliations:** ^1^ Department of Pathology and Laboratory Medicine, School of Medicine, University of North Carolina at Chapel Hill, Chapel Hill, NC, United States; ^2^ Department of Genetics, School of Medicine, University of North Carolina at Chapel Hill, Chapel Hill, NC, United States; ^3^ Lineberger Comprehensive Cancer Center, University of North Carolina at Chapel Hill, Chapel Hill, NC, United States; ^4^ Department of Pediatrics, School of Medicine, University of North Carolina at Chapel Hill, Chapel Hill, NC, United States

**Keywords:** global medicine, pediatrics, oncology, molecular diagnostics, genetics

In conversations with nearly every parent of a child with cancer, oncologists must acknowledge the uncertainty of whether a child’s cancer will respond to the treatment prescribed. Our prognostic abilities have improved in settings where molecular testing is available, but crippling uncertainty about treatment response remains, emphasizing the depth of biology that we have yet to uncover. In this Research Topic, we present articles that cover a range of research types, each relying on molecular diagnostic approaches, to work toward better tools and knowledge for clinicians and their patients.

For extremely rare tumors and presentations, case reports with literature reviews offer insights into natural history, prognosis, and molecular alterations. Pellegrino et al. demonstrated this through a case report of *malignant ectomesenchymoma*, drawing attention to the frequency of *HRAS* mutations (in this instance found by whole exome sequencing) in this biphenotypic tumor type with features of mesenchymal and neuroectodermal elements. In a similar type of report, Ogawa et al. described an unusual case of an *atypical lipomatous tumor*, with the diagnosis supported by the identification of *MDM2* amplification on fluorescence *in situ* hybridization. In both cases, molecular findings supported the final diagnosis. However, molecular results are not required to complete the diagnosis, and are not fully specific for each diagnosis, highlighting the challenge for pathologists to determine appropriate molecular evaluations for various pediatric tumors.

Another set of articles focused on molecular-based translational research, assessing potential prognostic markers and cancer susceptibilities. Braghini et al. presented research that builds on knowledge of potential therapeutic targets in hepatocellular carcinoma to explore similar pathways in *hepatoblastoma*. Their early *in vitro* data suggested that overexpression and activation of focal adhesin kinase (*FAK)* occur in hepatoblastoma, and that inhibition of this pathway can slow proliferation and induce apoptosis of cell lines. This early work must be followed by expanded research with larger cohorts of hepatoblastoma and *in vivo* models. Other efforts to explore new biomarkers of prognosis and targets focused on exosomes and telomere biology. Bhavsar and Morini, presented a review describing recent progress in understanding and exploiting exosomes in tumor biology. With a focus on *neuroblastoma*, the authors emphasized the growing literature utilizing exosomes as potential circulating biomarkers and therapeutic vehicles for precision tumor therapy and vaccination. Similarly, Burrow et al. summarized data on telomere biology as a potential prognostic marker with potential therapeutic implications. Using a highly sensitive rtPCR-based C-circle assay (CCA) to detect alternative lengthening of telomeres (ALT), they characterized the prevalence and clinicopathological association of ALT in *pediatric sarcomas*.

For the majority of cancer types, molecular testing is required for complete clinical classification to inform prognostic and therapeutic decisions. Even so, questions about the “what” and “how” of molecular testing for clinical care are complex. Which technical assay should be used with which computational algorithm? How can the global community commit to equitable access to molecular diagnostic tools? The last two articles in this Research Topic speak to some of these implementation challenges.

Skitchenko et al. highlighted the analytical challenges of nontargeted genomic sequencing. They assessed the germline predisposition to cancer of a child with *medulloblastoma*. They used whole exome sequencing results taken from two different analytical pipelines. Computational differences in these pipelines caused discrepant results that would impact clinical care. While their case description does not provide a solution to the challenge of implementation of computational tools, it sheds important light on the need for ongoing expert discussion to determine necessary validation steps for computationally heavy diagnostic assays.

Finally, Gastier-Foster et al. demonstrated the potential of the NanoString nCounter system as a targeted fusion detection approach for *acute leukemia* in Malawi. The authors showed that this technology can be utilized in a low-income country and that it would provide clinically important diagnostic information beyond the current standard of care testing. Their work is an important contribution to efforts to overcome diagnostic limitations in resource-limited settings. These findings also raise two critical challenges in the implementation of advanced molecular testing specific to low-resource settings: How should molecular tests be validated locally when the “truth” of the sample is not available? How do we consider the scalability and sustainability of a new test at the outset of clinical validation?

The collection of papers on this Research Topic shows exciting progress ranging from improved molecular description of exceedingly rare tumors to identification of additional biomarkers with potential for future clinical use to advancing the conversations about the clinical use of new molecular assays and analytical tools. Ongoing needs from the community of pediatric cancer researchers, pathologists, and hospital administrators include (1) continued identification of molecular biomarkers for risk stratification and identification of therapeutic vulnerabilities, (2) development of rational recommendations for clinical molecular testing for different tumor types, potentially with resource-adapted guidelines, and (3) a global commitment to implementation of molecular testing across different resource settings.

